# Insanity

**Published:** 1820-09-01

**Authors:** 


### INSANITY.

1. *Sound Mind; or, Contributions to the Natural History and Physiology of the Human Intellect.* By JOHN HASLAM, M.D. Octavo, pp. 192. London, 1819.

---

2. *An Inquiry into certain Errors relative to Insanity, and their Consequences; Physical, Moral, and Civil.* By GEORGE MAN BURROWS, M. D. F. L. S. &c. &c. &c. Octavo, pp. 320. London, 1820.

---

3. *On Mental Derangements.* By M. ESQUIROL, M. D. Physician to the Salpetriere, &c. &c.

[Art. FOLIE, Dict. des Sciences Med.]

---

" Huic ego vulgum
Errori similem cunctum insanire docebo." HOR.

---

THE union of mind and matter is a mystery that will never be solved by man; and consequently, the disquisitions on it will be interminable. The intimate connexion or absolute dependance of mind on matter, will for ever furnish the materalist with inexhaustible arguments for their identity; while the wide range of phenomena exhibited by the operations of mind, and which are totally inexplicable by the known laws of matter, will furnish abundant arguments for the Platonic Philosopher to vindicate the existence of that—

——————— Divinity that stirs within us,

And points out an eternity to Man!

We will not, therefore, involve ourselves in metaphysical speculations; but keep to the usual line of our policy, practical pathology and therapeutics.

Of the three works which lie before us, the two first afford us comparatively few materials for the construction of this article; Dr. Haslam's Essay being almost entirely composed of elementary and physiological facts and deductions, well known to every physiologist and philosopher; while the work of Dr. Burrows, though exceedingly important in its way, turns chiefly on the correction of certain moral, civil, and popular errors attending insanity, the Insane, and Lunatic Asylums.

A perusal of Dr. Haslam's Essay will convince any man that the author's *mind is sound,* his judgement correct, his perceptions clear, and his deductions legitimate. The style of his work too is of a very superior kind, and might be taken as a model for philosophical and medical disquisitions.

From Dr. Haslam's concluding chapter we shall condense some reflections, and exhibit some specimens that *may* prove interesting to the reader, and *must* prove honourable to the writer.

Dr. H. well observes, that a contemplation of every gradation of mental energy, from man to the maggot, impresses us with the evidence of appropriate contrivance and wisdom. The mental endowments, however, which animals possess, keep them stationary. They cannot comprehend; and if they did, they want the means to communicate, consequently every attainment perishes with the individual.

"As they have not been endowed with the capacity to numerate, they can experience no solicitude for the past, nor apprehensions for the future. In the Grammar of Animals, the present is the only tense;* and to punish them for the faults they had formerly committed, would be equally absurd and tyrannical. They are not the creatures of compact, and being unable to comprehend the nature of institutions, and obligation of laws, they cannot be responsible agents." 187.

The above truths only go to prove that animals are not amenable to human laws; and the conclusion is, that a future state of existence for them is highly improbable—for *there* too, they could not be justly punished or rewarded.

The features of the human mind are very differently shaped, "and strongly indicate an ulterior destination." Man is rapidly progressive, his mind becoming opulent from the intellectual treasures of his ancestors, which he bequeaths, in turn, to his posterity.

"Had he, like animals, been gifted with intuitive wisdom, the donation would have been so perfect, as to render instruction superfluous; and such endowments would have diminished the measure of his responsibility. The freedom of his will, by which is to be understood the impulse of reason, not the blind dictates of appetite, nor the sallies of tumultuous passions, renders him amenable." 190.

Man alone can repent; retracing the acts of former commission, and resolving on amelioration for the future.

"In proportion to our love and estimation of justice, we must be satisfied that, under the present forms of government, it is but im-

* This is going rather too far. The bee in constructing his cell, and the beaver his honse, certainly appear to know something of the *future* tense, as well as the present. *Rev.*

perfectly administered: the rewards and punishments in this life will ever be blended with the hopes and fears, the interests and passions of our species; and there is much of evil, which human sagacity cannot detect. When we consider the attributes of Deity and the nature of Man, we can never be induced to conclude that the tribunals of this world are the courts of final retribution. Man bears in his intellectual construction the badge of moral responsibility; and consequently, the germ of future existence: and the only incentive that can urge him to the advancement of science, and the practice of virtue, is the reward that Revelation has unfolded." P. 192.

From these sublime and momentous reflections, we must descend to the concerns of our frail and perishable existence, in this sublunary scene. We come, therefore, to the work of Dr. Burrows.

Our author justly conceives, that the scholastic dogma of insanity being a disease of the *mind*, as an immaterial essence, has been very prejudicial, by directing the practitioner's attention entirely to moral remedies, where the predicate, he thinks, may be safely hazarded, "that he who relies, singly, on moral means, will be as surely disappointed, as he who resorts to the art of medicine only, for the cure of insanity." 7.

Dr. Burrows seems to have good reason for believing, that the curability of insanity is considerably under-rated by the world at large. In short, the *cures* of insanity are as carefully concealed in families, as the disease itself. No eclat attends them.

"The physician by whose skill they are accomplished is never mentioned. There is only the darker side of the picture presented to the public view; and consequently a reproach, severer than deserved, attaches to the healing art." 10.

Among the prevailing errors regarding insanity, our intelligent author ranks, 1st. its difficulty of cure; 2d. that insanity is an increasing malady; 3d. that insanity is an exceedingly prevalent malady. This leads him to open the body of the work with a discussion on the above topics.

1. *Is Insanity curable; and in what proportion?*

Although the records of Lunatic Asylums are very defective, yet, from numerous returns which our author has collected, with great industry and expense, from several private, and all the public establishments for the insane in England and Scotland, Dr. Burrows concludes, that not only is insanity a curable disease, but "that a very large proportion of lunatics, under a proper system of management, actually recover." 36.

*The second question is, whether Insanity be as susceptible of cure as other maladies?*

Without discussing the question whether insanity, like most other diseases, has its access, crisis, and decline, this much is certain, that "the difficulty of cure is more or less, according to the time which has elapsed since the incipient attack, or the complication of mental with bodily complaints." We apprehend that Dr. Burrows would have been more correct to have said "*moral with physical,*" than "mental with corporeal;" since all acknowledge now, that insanity is a *corporeal* disease, either of function or structure. But this is of no consequence.

Now it unfortunately happens, as Dr. Burrows well observes, that the approach of insanity, though generally perceptible, especially to strangers, is rarely remarked by relations; but is construed into nervous irritability, or eccentricity, or any thing rather than the right. The complaint is suffered to proceed till some terrible exacerbation of delirious fury or despondency ensues, and the malady is thus confirmed in one whose intellects, very probably, might have been preserved, had timely aid been administered. Thus, "did mental derangement experience the same prompt attention as most other complaints, it is impossible to judge how much more favourable the results might prove." But, as Dr. Burrows justly observes, the reverse almost always obtains; and consequently, insanity more frequently degenerates into a chronic or continued type than any other disease, and has not the same advantage from the means of cure.

In addition to the above obstacles, there is too often opposed to the exertions of the physician, the influence of pride, suspicion, deception, and alas! the avarice, or other interested motive of friends, who very often object openly to the prescription of remedies. Yet, under all these disadvantages, we have incontrovertible evidence how great a proportion of lunatics recover, as the records of public and private Asylums testify. In 1798, Dr. Willis stated, in the House of Commons, that nine cases out of ten of insanity recovered, if placed under his care *within three months from the attack.* Doubts of his veracity were then and afterwards entertained; but few men ever possessed the essential auxiliaries to success which Dr. Willis did.

"We now see that, in situations, perhaps less promising, eight in ten, and even six in seven *recent* cases are reported, and believed to have actually recovered." 44.

In La Salpetriere, at Paris, the proportion of cures of recent cases, was, 1806-7, according to Dr. Carter, almost as high as that of Dr. Willis; and, according to Dr. Veitch's official statement to Parliament, nearly two in three of the recent cases were discharged cured, while only five out of one hundred and fifty-two old cases recovered. In Dr. Burrows's own practice, of about three hundred cases, the proportion of cures exceeds any which is here stated; being ninety-one per cent. in recent, and thirty-five per cent. in chronic. Upon the whole, our author thinks he is justified in concluding, that insanity is fully as curable as the general run of other diseases.

The next question is—"*Is Insanity an increasing malady?*"

Mental derangement has been truly designated the "vice of civilization;" for the more polished and artificial the people are, the more prone are they to insanity. In Spain, where the inhabitants are more characterized by primitive manners, especially temperance, mania is said to be comparatively rare; while the unsophisticated Aborigines of North and South America, are reported by Rush and Humboldt to be wholly exempt from this irritation.

All the physical causes of insanity Dr. Burrows considers to be *extraneous* or artificial, except one; to wit, hereditary predisposition, or that peculiarity of organization transmitted from parent to progeny, and "which alone can be rationally supposed to have a progressive operation in augmenting the number of the insane." To this we cannot entirely subscribe. Dr. Burrows, in the preceding page, acknowledges that the more polished and artificial the people, the more prone are they to insanity. It follows, surely, from this, that the progress of civilization augments the *causes* of insanity, and consequently the number of the insane. That civilization, refinement, luxury, and effeminancy, are, at this moment, on the increase, we believe few will be inclined to deny. We conceive too, that the more *thinking* a nation becomes, the more prone will it be to mental derangement. Learning is every hour increasing throughout the lower orders of society, and we all know—

"That shallow draughts *intoxicate the brain;*"

nor is it likely the aforesaid classes shall drink so deeply at the fountain of knowledge as that it shall "sober them again." When we come to Dr. Esquirol's work, it will be seen how powerfully scepticism and materialism have tended to destroy the happiness of the French people, and conduced to insanity and suicide. It is a melancholy truth, that irreligion and scepticism are making rapid strides in this country, and the consequences will be doubtless the same as in France. It is from a general view of these circumstances, rather than from the numerical returns of Lunatic Asylums, that we would be led to the conclusion, that insanity has increased and is increasing.

The following extract from a private letter of Dr. Halloran's to a physician of the metropolis, shews that in Ireland, at least, insanity is on the increase.

"With us it is but too obvious that this malady is still greatly on the increase; and the misfortune is, that we have not even yet seen the worst of it. The desperate break-up of property here, amongst the speculative farmers, and *middle men*, as they are called, who, for a series of years, were in the enjoyment of affluence, is beyond all belief. To these, in particular, now reduced to actual poverty, in numerous instances, may be ascribed the extraordinary run of maniacal cases which, in fact, are daily offering; and who, on feeling their individual share of the general crash, are unable to bear the reverse of circumstances to which they are exposed. In order to drown care, therefore, they become an easy prey to the effects of drunkenness, want, and disappointment combined."

To such an extent has the misfortune arisen, that the public Asylum at Cork is now overloaded with fifty cases more than it is calculated to hold, and the Grand Jurors have voted a sum of £.4400 to be laid out, under Dr. Halloran's superintendance, in the construction of increased accommodation for the insane.

This, by the bye, is a tolerable proof of the respect in which the professional and private character of Dr. Halloran is held by his fellow citizens, though one of our London hyper-critics accuses him of ignorance and materialism!

Dr. Burrows enters into a long and critical examination of the registers above mentioned, the aggregate of which exhibit an evident increment of the disease. But we cannot follow our able author in this scrutiny. He has plainly proved that these returns are fraught with error, and exceedingly liable to be influenced by various circumstances that render them weak authority. Still, as we said before, we look to the etiology of insanity, rather than numerical tables, for evidence of its progressive increase.

In the question of the *comparative* prevalence of insanity in this, and other countries, Dr. Burrows has brought forward many important documents, and made some excellent observations. He has clearly proved, that *suicide* is infinitely more prevalent in Paris, Berlin, Copenhagen, and probably many other capitals, than in London; and thus rescued his country from a common, though unjust, accusation. This section is concluded in the following words.

"But enough, surely, is advanced, to refute the general conclusion, that insanity is extraordinarily prevalent, and that it exceeds in England." 105.

In the next section, indeed, our author attempts to prove, from certain official returns, that insanity has actually decreased within the last few years, which he attributes not to any abatement of the *causes* of the disease, but to a more successful *practice* pursued. That the treatment of insanity, both moral and physical, is improved of late, we are ready to grant; but we are somewhat sceptical, as to the inference which our author has drawn on this point. We must pass over some sections on the state of Lunatic Asylums, and on the condition of the epileptic, fatuous, and idiotic, as not coming within the objects of our Review, though highly creditable to the author and interesting to the public.

The 9th section embraces the question—"*Is Religion a cause or an effect of Insanity?*"

Dr. Burrows quotes the opinion of the Chancellor de l'Hopital, "that religion has more influence on mankind than on all their passions combined." Of this truth, he thinks the whole world is an illustration.

"And as there is no single passion that may not be excited to an excess inducive of mental derangement; so we may readily believe that religion, which influences the internal man more than the passions collectively, may be a cause of insanity. On the other hand, there is no doubt that a lunatic may imbibe a religious, as well as any other hallucination; and yet be insane from a cause very contrary to religious. In the one case, however, it is a cause; in the other, an effect. Now, a great source of error seems to arise from the confounding of this necessary distinction." 173.

We believe that it would be more correct to call it fanaticism than religion, when it produces insanity. The following sentiments, from such a man as Dr. Burrows, will be read with interest in the present times of scepticism and irreligion.

"Religion, it must be acknowledged, is the very essence of humanity. Without it, man has no guide but his passions—no law but his will. Even savages have some notion of a Deity, or a future state: and although it be not always a God of Mercies they adore, yet, divest them of the sense of a superior and presiding Power, and the character of the people would sustain a material change; and, perhaps, for the worse. What follows, then, when scepticism and infidelity reign, where Christianity once shed its pure and benign influence? The human mind having lost that prop which was its stay in the hour of need, chaos ensues; despair succeeds to hope; and reason, which establishes man's supremacy on earth, is overthrown. Here insanity supervenes on the defect of religion,

As a cone inverted; so, we may be assured, is the state of morals where religion has been extinguished: it is a fabric without a foundation; and there insanity will emanate and most exceed." P. 178.

Dr. Burrows thinks that, were the remote cause of insanity, when marked by religious exaltation or despondency, to be traced, the lunatic would be found a convert from his original to some new faith—" and that the aberration was usually developed during the transition."

Our author illustrates the above position by five or six very apposite examples, which came within his own cognizance. Indeed, the whole of this section is executed in Dr. Burrows' best style of strong and forcible reasoning. At page 218, there is a passage which we deem worthy of extracting, for the benefit of our readers.

" Were I to allege one cause, which I thought was operating with more force than another, to increase the victims of insanity, I should pronounce, that it was the overweening zeal with which it is attempted to impress on adolescence, the subtle distinctions of theology, and an unrelenting devotion to a dubious doctrine. I have seen so many melancholy cases of young and excellently disposed persons, of respectable families, deranged from either ill-suited or ill-timed religious communication, that I cannot avoid impugning such conduct as an infatuation, which, as long as persevered in, will be a fruitful source of moral evil. The old Romans knew human nature better: they had a law which forbad any person entering upon the sacerdotal office before the age of fifty. This was to prevent theological discussions before an age was attained when a bad effect was not to be apprehended." 218, 219.

Dr. Burrows objects to the commonly-received opinion, that "religious insanity," as it is called, is incurable, or at least, extremely difficult of cure. Whenever he has been left to follow his own judgment, however deeply the mind was imbued with religious delusions, he has never, in recent cases, found particular difficulty in curing the patient.

The subject of the 10th section naturally arises out of the preceding:—" the efficacy of religious instruction of lunatics." We entirely agree with Dr. Burrows, that before religious instruction, in any form, be attempted, an intimate knowledge of every patient's state of mind, and of his former and present opinions, should be acquired; consequently, that no minister can assume this office of instruction, unless he have constant intercourse with the patients, and be well acquainted with each. No man, except a zealot or fanatic, will question the truth of the following passage.

" Suppose a certain number of lunatics were selected, whose cure, it may be thought, religious instruction will facilitate. Is it not clear, that the spiritual admonitions which may be adapted to one, may be a source of irritation to another? For where men who are called sane, are so exceedingly tenacious about the mere forms and ceremonies of worship, and are thence impelled to acts little short of madness; how can we imagine, that, among a number, some insane, some weak of intellect, and some not confirmed yet in judgment, offence should not be taken, if the doctrine or rites most consonant with each patient's notions, be not preferred? Without, therefore, the utmost precaution, it is not difficult to determine, that the introduction of spiritual subjects must be dangerous and often injurious." 227.

There are doubtless, as Dr. Burrows observes, many lunatics to whom religious instruction would be extremely useful; but it is evident that this must be exhibited *individually*, and adapted to the particular case. It is extremely problematical if any discourse, however carefully composed, would ever suit a lunatic *audience*.

"It results, that religious instruction must, in the first instance, be tried as an experiment: the only safe way in which it can be essayed, is by a previous personal examination of each patient's state of mind and feelings; and if pronounced in a fit frame, there can be no question, that the inculcating of the simple and benign precepts of Christianity, will not only be found an efficacious auxiliary to the restoration of a sane understanding, but to the subsequent preservation of it. For true religion yields to the afflicted—relief; to the sinner—hope; to the repentant—forgiveness; and to all who possess a sound mind, and believe, is "the source of light and life, and joy and genial warmth, and plastic energy." 232.

The remainder of Dr. Burrows' work is principally taken up with "suggestions respecting legislative regulation of Lunatic Asylums, &c." and tables of official and other reports from various Institutions, public and private, for the reception of insane persons. These we cannot enter upon, in a medical review; but refer to the work itslf, which does great credit to Dr. Burrows's industry, zeal, and well-known talents.

From his strictly medical work on Insanity, which he has long promised to the profession, and which he still promises, we anticipate a fund of important and useful observations, an account of which we shall not be slow in communicating to our readers. We may remark, in closing this article, that the author has candidly apprized the public that the work is not to be considered as a contribution to *medical science*, in its strict sense; it is consequently written in a familiar, but certainly a neat, and even in some places an elegant style, perfectly appropriate to the subject of the volume.

And now we must take up Dr. Esquirol's Essay on Insanity, which is more adapted to the nature and design of our Review than the two works of our respected countrymen, Drs. Haslam and Burrows.

Among the leading articles in that great national, but unequal work, the DICTIONNAIRE DES SCIENCES MEDICALES, Dr. Esquirol's paper on Insanity, holds a distinguished rank, not only on account of the talent, but the extensive experience, and accurate observation of its author. The article occupies eighty pages of the Dictionary, and has cost us considerable labour to translate, analyse, and condense it, within the limits of this Review. We hope, however, that we shall render our professional brethren, in this country, some service, by diffusing among them the observations on insanity of an illustrious foreigner, though at the expence of some midnight vigils on our parts.

---

§ I. The symptoms of insanity relate to an alteration in the thinking faculty—a subversion of the moral affections—lesions of function in the organic life. The sensations of the insane are perverted, and they appear the sport of erroneous impressions. The sense of *sight* evinces a thousand illusions. The maniac often knows not his friends or relations, frequently mistaking them for strangers or enemies. A great number of insane imagine they *hear voices,* which speak distinctly to them, and with which they hold conversations. These *voices* pursue them, and harass them, by day and by night—in public and private—in their walks—in their travels and in their closets. The sense of *smelling* does not escape these perversions.

"*Case in Illustration.* A lady, 27 years of age, in the last stage of phthisis, perceived in her room an odour of charcoal. She immediately conceived that there was a design against her life. She left her lodgings, but the *fumes of charcoal* incessantly pursued her till her death."

It is not uncommon for the *taste* to be perverted at the commencement of insanity, generally from derangement of the stomach, the perversion disappearing with the cause which produced it. How frequently do we see the alienated deceived in respect to the size, the form, and the weight of things around them. The greater number of them become unhandy in all mechanical occupations, music, writing, &c. The *sense of touch* having lost its peculiar power of correcting the errors of the other senses.

These perversions sometimes affect but one sense, often two—more rarely three—but occasionally all. Errors of hearing and sight, however, are by far the most frequent of the perversions. With these derangements, we also find combined, a *multiplicity* of sensations—a superabundance of ideas, and a versatility of resolutions, produced without order, object, or end. This exuberance of thought permits not the insane to fix his attention long enough on each sensation, and each idea, so as to separate the false from the true—consequently, he becomes incapable of comparing and abstracting. The result is, a fleeting delirium, [délire fugace] the object of which is perpetually renewed, in every imaginary form. More generally, however, there is one predominant idea or train of thought, on which the attention is exercised with such intensity, that all others are excluded, or, as it were, swallowed up. This may be termed *Monomania*.

In other cases, the sensations appear to be so weak as to leave little or no impression on the sensorium. The patient remembers only things long past, and no new ideas are formed. Such is idiocy.

In some cases of mental alienation, the person seems beyond the empire of the will, and is no longer master of *his own* determinations. He appears directed by an irresistible impulse, to acts which he himself abhors. One person will feel condemned, as it were, to silence and inaction; another will walk, talk, dance, sing, or write, without the power of stopping. Others again, commit acts of fury, at which they shudder the next moment. All the phenomena of insanity show the vast influence of the *passions* in this affliction. They are always *impetuous*, whether they be of the gay or the sombre cast, in mania, monomania, and melancholia.—In idiocy, they are only such as are founded on the primary wants and impulses of man—as love, anger, jealousy.

In mental derangement, the sense of delicacy is obliterated; and people of the finest previous feelings, will deliver themselves up to the most indecent or culpable actions, without the consciousness of impropriety.

Pusillanimity is a remarkable trait in the character of the insane. They are timid, distrustful—suspicious—never satisfied with their present condition, but always desirous of being where they are not. They have no care for the future—all their anxiety is for the present moment. It is this discontent of mind which detaches them from their parents and friends, and causes them to hate the most, those whom they before most cherished. There are a few exceptions, indeed, where the insane still preserve an inviolate affection for those whom they previously loved or esteemed: but such affection is without confidence in the objects, to whom they will give no ear in respect to precepts or advice. This alienation from friends is one of the most constant and pathognomonic traits of the malady.

The return of the moral and social affections, within just bounds, the desire of seeing their children, parents, or friends, and of resuming their wonted habits or employments, afford the best hopes of recovery; and *vice versa.*

§ II. In respect to *physical* phenomena, we see the vital powers of the insane often exalted to a degree that enables the maniac to resist the influence of very powerful external agents. But this exaltation is not so *general* throughout the system as people imagine. If there be greater force or vital power in one class of organs or parts, there will be a deficit in some others.—Among the physical phenomena of insanity, few are more constant or remarkable than want of sleep, and that peculiarly disagreeable odour from the body, as well as the excretions of the patients, which impregnates the clothes and bedding. Some are devoured with a burning internal heat; most have a voracious appetite, and constipation or other irregularity of the bowels. A great proportion are afflicted with *pain* in some organ or part, especially the head, the chest, or the abdomen, which the insane generally attribute to the malevolence of their enemies.

From the preceding and many other phenomena, then, mental and corporeal, we may fairly conclude that, in insanity, the vital powers are deranged; the senses impaired; the faculties of perceiving, comparing, and associating the ideas, disturbed; together with more or less lesion of function in the organic life. Such are the *facts,* without any mixture of hypothesis, and without attempt at explanation. Nevertheless, the following hints may be practically useful.

A young man conceived himself at court, and surrounded by courtiers. He prostrated himself at the feet of the supposed sovereign, and became enraged when he saw the servants take liberties with his imaginary king. M. Esquirol caused a bandage to be placed over his eyes for two days, during which the delirium entirely ceased; but on removing the bandage, the hallucination returned. Reill states, that a woman conceived she saw spectres, and fell into a convulsive delirium. Her maid servant placed her hand over her mistress's eyes, when the latter immediately cried out, "I am well," the spectres having disappeared. These hints are worthy of attention, especially in monomania. The insane, on recovering, preserve a distinct recollection of all that past during the hallucination.

§ III. M. Esquirol classes insanity in the following order.

A. Monomania or melancholy; in which the hallucination is confined to a single object, or to a small number of objects.

B. Mania; in which the hallucination extends to all kinds of objects, and is accompanied by some excitement.

C. Dementia; wherein the person is rendered incapable of reasoning, in consequence of functional disorder of the brain, not congenital.

D. Idiotism congenital, from original malformation in the organ of thought.

M. Esquirol then presents four figures drawn from life, illustrating these four classes of insanity, with graphic sketches of the patients.

"The first, or example of melancholia, is a young woman, 23 years of age, who was brought into the Salpetrière in July, 1812, perfectly taciturn, and desirous of being left quietly in her bed. Great difficulty was experienced in persuading her to take food; but the effusion of cold water conquered her resolution. During the four years that this woman has remained in the hospital, she has scarcely uttered a syllable. She must be taken out of bed, and when placed on a seat she remains in the same attitude the whole day afterwards; the head reclining over the left shoulder; the arms crossed; the eyes fixed. The food is put to her mouth, and she eats without changing posture. When placed in bed, she muffles herself up, and remains in the same position the whole night. She is meagre, her skin brown; her hands and feet often of a violet colour; pulse slow and feeble; menses very irregular; constipation obstinate.

"The next plate represents *mania furibunda*. The unfortunate subject of it is a married woman, 55 years of age, who entered the Salpetrière, April 1814, in the state above mentioned. She is of tall stature; hair white and bristled; eyes blue, sparkling; haggard; physiognomy very changeable; skin pale; emaciated.

"It appears that some losses in business, injurious reports, and domestic troubles, gave origin to the complaint. Her hallucination was general; she abused all the world, was excessively violent, and in constant agitation. Warm and cold baths, opiates in large doses, and various other means were tried, in vain. Nothing but the straight waistcoat could secure her. On the 4th of January, 1815, one year after the commencement of the malady, the weather being very cold, she suddenly expired, without any premonitory symptom indicative of such an event. Anatomical investigation could discover no trace of altered structure in any part of the corporeal fabric.

"The third plate presents the profile of a female 70 years of age, who, after having passed several years in a state of mania furibunda, is fallen into that of DEMENTIA or idiotism.

"The hallucination of this patient corresponds with her advanced age, and the long duration of the complaint. She preserves a few ideas, which still savour of pride. She believes herself the daughter of Louis XVI. but otherwise there is no coherence; no memory of recent transactions; no hopes or fears, desires or aversions. She is calm, peaceable; sleeps well; eats without voracity; and appears perfectly happy.

"The fourth plate exhibits the profile of an idiot; a young woman, 21 years of age, who has been in the Salpetrière three years, without any change. Her head is large and irregularly shaped; the forehead high and prominent, so that the facial angle is more than 90°. She eats voraciously, and without discrimination; passes all evacuations involuntarily; menses regular and abundant; walks little; all her movements convulsive: in short, she is a perfectly helpless infant; insensible to every thing around; heat, cold, rain, or even her own internal feelings. She can only utter the words '*papa and mama,*' which she frequently repeats. No history of her complaint could be procured."

Having now sketched an outline of the symptomatology of insanity, we proceed to an interesting division of the subject; its ætiology.

§ IV. *Causes of Insanity.* These are very numerous, both moral and physical. Climate, seasons, age, sex, temperament, profession, modes of life, political situation, civilization, public manners—all exercise an influence in the production of this calamity.

*Climate.* Insanity does not abound in hot climates, but in the temperate; especially those which are subject to great atmospherical vicissitudes. The influence of climate on this disease, however, has been exaggerated. Thus Montesquieu attributed the great number of suicides in England to the foggy state of its atmosphere; but we shall trace the circumstance presently to a more powerful cause.

Insanity is evidently *endemic* in some regions. In marshy countries *idiotism* prevails. *Cretinism* is endemic in the gorges of the Mountains. A member of the Institute informs us that the disease was found only where the soil was *calcareous*.

*Season.* Extremes of heat and cold, in Europe, conduce to the production of insanity. We see the effects of coups de soleil. The French soldiers, on first going into the hot vallies of Spain, were much affected with insanity; and the same was the case in the disasterous retreat from Russia. Many of the officers and soldiers became insane, the disease being first acute, passing suddenly into the chronic state. In those who died, the dura mater was found very much thickened.

"The insane are greatly disturbed and exasperated by any sudden atmospherical commotion. About the Equinoxes, each Lunatic Asylum presents a scene of louder uproar, and requires stricter discipline, than at other seasons. From tables kept at the Salpetrière, during the last nine years, it appears that the admissions were most numerous in the months of May, June, July, and August—less so in September, October, November, and December; but least of all, in February and March. The influence of the seasons on the *march* of the disease bears analogy to the above rule."

In respect to *Lunar* influence M. Esquirol cannot confirm the long prevalent opinion. He observes, that the insane are certainly more agitated about Full Moon; but so are they also about day-break, every morning. Hence, he conceives, the *light* to be the cause of the increased excitement at both those periods. Light he asserts, frightens some lunatics, pleases others; but agitates ALL.

Some authors have ascribed an occasional *epidemic* character to insanity; as Stegman and Sydenham. It is certain that, in some years, independently of all moral causes, insanity is more prevalent than in others.

*Age.* Infancy appears to bid defiance almost to insanity, unless there be some congenital malformation, or idiotism be induced by convulsions or epilepsy. Nevertheless our author, and others have met with cases of mania arising in very young people. It is about the age of puberty, however, that the causes of insanity begin to operate powerfully on youth. The disease, at this epoch, of course, is characterised by rapid march, and height of excitement; in adult age, it is more chronic. In early life, it is more commonly complicated with affections of the abdominal viscera, and is relieved by the development of hæmorrhoids, and by critical discharges from the bowels. At a more advanced age, with paralysis and apoplexy; and is of more difficult cure.

*Sex.* Upon a comprehensive comparison, it has been found that there is no other disproportion among the insane of both sexes, than among the sane population in general.

*Modes of Life.* Intense study, especially where the efforts of the mind are directed more exclusively in one channel, predisposes to insanity. But great genius, and well regulated learning, have no tendency of this kind. If a man, however, meditates incessantly on a particular subject, metaphysical or speculative, all his physical and moral faculties are absorbed, as it were, in the pursuit; his health is neglected; the epigastric viscera become deranged, with torpid chylification, the result of bad digestion; the secretions are depraved; the functions of the skin impeded; and hypochondriacism or melancholy steals over the devotee of letters and meditation. If these excessive meditations and studies take the channel of religion or fanaticism, then there is great danger that the seat of reason may sooner or later give way. Various epochs have been remarkable for the prevalence of insanity; as the times of the Crusades: the Calvinistic and Lutheran disputations, revolutions, &c. Liberty and reform gave a strong impulse to insanity in France, "and it is remarkable (says M. Esquirol) that the character of the insanity varied and corresponded with the different storms which burst over our country." *"Il est remarquable que les folies qui ont éclaté depuis trente ans, ont eu pour caractère celui des différens orâges qui ont troublé nôtre patrie."*

The progress of civilization, too, by laying us so much at the mercy of the vicissitudes of fortune around us, and thereby rendering us morbidly sensible to every occurrence, predisposes astonishingly to insanity. Hence the sceptre, the court, the palace, the high military command, the exchange, and riches themselves are no protection against this deplorable evil!

Sedentary habits, intoxication, excess in venereal and solitary pleasures, all predispose to, or even excite insanity.

"La masturbation, ce fleau de l'espece humaine, est plus souvent qu'on ne pense, cause de folie, surtout chez les riches."

A twentieth part of the insane females admitted into the Salpetrière are previously prostitutes. The following melancholy picture presents an awful lesson to the world at large!

"Religion (says our author) no longer obtains, but as a mere ceremony, on certain solemn occasions. She no longer carries consolation or hope to the bosom of the wretched! Religious morality no more serves as a guide for reason, in the rough and intricate path of life. Cold-blooded selfishness has rooted out every source of feeling; and there is no where to be seen either domestic affection, respect, love, obedience, or reciprocal dependance. Every one lives for himself; and the ties, which used to connect the present with future generations, are dissolved."

"La religion n'intervient que comme un usage dans les actes les plus solennels de la vie; elle n'apporte plus ses consolations et l'éspérance aux malheureux; la morale religieuse ne guide plus la raison dans le sentier étroit et difficile de la vie; le froid egoïsme a desseché toutes les sources du sentiment; il n'y a plus d'affections domestiques, ni de respect, ni d'amour, ni d'autorité, ni de dependances reciproques; chacun vit pour soi; personne ne forme de ces sages combinaisons, qui liaient à la generation future les generations presentes."

Alas! if these be the fruits of knowledge, it may be well said that "'tis folly to be wise."

Great as the horrors of the revolution were, M. Esquirol considers their influence in producing insanity as nothing, compared with the corruption of morals and manners, public and private, resulting from the doctrines of materialism.

*The Passions.* The first wants of our nature relate to the preservation of the individual, and the reproduction of the species. They are so strong and universal, that they may be termed instincts rather than passions. These last are the fruits of developed intelligence and advanced civilization. Infancy is free from the passions, and, consequently, from insanity. As puberty and manhood advance, the instincts come forth in all their strength, and afterwards the *factitious passions* take their ascendency; as self interest, ambition, pride, avarice, &c. Now it is, that insanity breaks out, in all its varied forms and characters.

The passions and emotions most productive of this complaint are, love, fear, fright, rage, ambition, reverses of fortune, and greatest of all, *domestic chagrin*, or family dissention, which comprises all the miseries to which poor humanity is liable in this world!

The exhilarating passions or emotions are rarely the cause of insanity. Excessive joy will destroy life, but not reason. Those who become insane, after the sudden acquisition of riches, may attribute the disease to the altered modes of life which result, together with the generation of new passions and desires, rather than to the first emotions of joy on the event.

A signal source of insanity, as M. Pinel observes, is the struggle between the principles of religion, morality, and education, on one side, and the passions on the other. This combat in the breast of man often ends in the loss of reason!

"Moral frequently combine with physical causes in producing mental derangement, especially in women. A young lady, under the menstrual movement, was frightened by a peal of thunder. The catamenia were suppressed; the head became deranged, and reason did not return till the menses were re-established some months afterwards. Another young woman had a safe accouchment. On the seventh day she was suddenly and unexpectedly reproached, in severe terms, by her father. The lochia and the mammary secretions were suppressed; mania furibunda followed, and required six months for its cure. This *combination* of moral and physical causes, in fact, is much more commonly the origin of insanity, than either of them singly. But the moral causes predominate far, in number and force, over the physical. It is the latter class, however, that comes more immediately within the province of medicine, and to which we shall now direct our attention."

*Hereditary Organization* is one of the most common physical causes of insanity, especially among the rich, where it is observable in every *second* case; while among the poor, the proportion is one in six. Children born before the commencement of insanity in the parent, are much less liable to the disease than those who are born afterwards. This unhappy predisposition is painted in the physiognomy, the exterior configuration, the ideas, the passions, and the habits of the offspring; and it is called into action by much slighter causes than would otherwise be necessary, where there is no hereditary taint. In short, it is perfectly analogous to the congenital predisposition to gout, phthisis, and some other hereditary diseases. This should always be borne in mind, in the education of children, whose parents were insane, and who should be placed as far as possible out of the reach of exciting causes of the disease. Hereditary insanity is not incurable on that account, though the cure is more difficult, and relapse more probable than under opposite circumstances.

The primary cause of insanity is often stamped on the tender fabric of the body, in utero, during lactation, or in the first dentition. Numerous were the women who, during the storms of the revolution, brought forth children, in whom the slightest causes produced insanity. Falls, or blows on the head, during infancy, frequently predispose to, or even excite mental derangement at some future period. Interruptions and irregularities of the menses were traced as the causes of insanity, in the proportion of about one in six; and the return of the catamenial period is, at all times, an epoch of irritation, in female insanity. M. Esquirol was led to attribute the origin of the disease, in many instances, to suppression of leucorrhœa in women, and the hæmorrhoidal discharge in men.

*Parturition* is a very frequent cause of mental alienation. Out of six hundred females in the Salpetrière, and M. Esquirol's private asylum, fifty-two dated the origin of their complaints at this epoch. Conception and pregnancy too, he believes, are occasionally the cause of this affection. He saw a young female who had a first attack of insanity immediately after marriage, a second attack at the period of conception, and a third attack during the second pregnancy. Each paroxysm lasted about a fortnight. Mental derangement occasionally takes place during lactation, generally with a preceding suppression of the milk.

Sudden suppression of the cutaneous transpiration is no uncommon cause of insanity; and it is in this way that atmospherical vicissitudes conduce to its production. A man was subject to copious sweats about the head. He was advised to wash himself with cold water. The perspiration was suppressed; but insanity became established. A young man waded across a rivulet while freely perspiring. He had a rigor on going to bed, and immediately afterwards turned maniacal.

Fevers not unfrequently leave a predisposition to insanity, which may not be excited into action till some years afterwards, when the fever is forgotten.

The sympathetic irritation of accumulations in the stomach and alimentary canal, has induced this disease, as also worms. A great number of chronic affections, as well by their inconsiderate removal, as by their spontaneous metastasis, have ended in mania. Epilepsy, in particular, is a fruitful source of mental derangement. Of three hundred epileptics in the Salpetriere, more than half are insane! Hysteria and hypochondriasis too often degenerate into the disease under consideration. The abuse of mercury and opium is occasionally the source of this complaint.

§ V. *Progress of Insanity.* The causes of insanity do not always act *directly* on the brain; more frequently, on the contrary, they are preying on some organ at a distance, the nervous, sanguiferous, or lymphatic systems; the digestive organs, or the organs of generation, being the primary seats of the evil. The *exciting* causes of insanity, moral and physical, sometimes produce the disease *suddenly*, especially in those who are strongly predisposed; but, in by far the greater number of cases, certain functional derangements have been going forward for years before the mental hallucination is developed; such as convulsions, head-aches, spasms, constipation, &c. On the other hand, in the intellectual system, we see ebullitions of the passions, or excentricities of temper, long precursory of the final overthrow of reason.

Insanity is either continued, intermittent, or remittent. The remissions of the disease offer some curious anomalies. A person will pass three months, for instance, in a state of melancholy; the three following months in maniacal excitement; and then three or four months in a state of fatuity; and so successively, with more or less regularity. There are others who only experience paroxysms of excitement at certain hours of the day, days of the week, or even seasons of the year, the intervals being passed in a calm and quiet hallucination.

Insanity appears sometimes to terminate critically, like a a fever. A certain change in the countenance, with sense of general lassitude; sleep; appetite; softness of the skin; freedom of the secretions and excretions; and a return of moral feeling, are indicative of approaching recovery. This will be perfect if, with the return of reason, the patient resumes his usual affections, habits, and general character. But if, on the other hand, the functions of the organic life are re-established—if the sleep, the appetite, the secretions, and the excretions come back to their usual healthy standard, without a corresponding sanity of mind, the disease is likely to pass into a chronic state, or fatuity itself.

§ VI. *Curability of Insanity.* From an accurate survey of the records of Lunatic Asyla, we are authorised in concluding, that cures are effected in about one in three cases of insanity. The probability of cure, in individual cases, depends greatly on the previous duration of the disease. M. Esquirol has constantly observed a marked *remission* in the course of the first month, after which it generally passes into the chronic state. It is in the *first* month, therefore, that we have the great probability of success. Physicians are divided in respect to the medium curable period of insanity. Into the Salpetriere, in ten years, [1804 to 13] were received two thousand eight hundred insane; of whom seven hundred and ninety-five were ultimately pronounced incurable, in consequence of age, fatuity, or complicated diseases, as epilepsy, &c. Six hundred and four were cured in the first year; five hundred and two in the second; eighty-six in the third; forty-one in seven succeeding years. From these facts, we may conclude, 1mo. that, in the two first years of insanity, the greatest number of cures are performed; 2do. that the medium curable period is somewhat less than a year; 3tio. that after the third year, there is not above one in thirty cured. It is true there are a few rare instances of restored reason after a period of many years hallucination; but these cases are more calculated to prevent despair than inspire hope. The most favourable epoch of life for the cure of insanity is between the age of 20 and 30. After 50, there is little chance. Mania is more frequently cured than melancholia or monomania. Idiotism and the fatuity of old age are absolutely incurable.

There is a proportion of the insane who can only be restored to a *certain degree* of sanity. These retain a morbid and excessive sensibility to all the causes, moral and physical, which produce the disease. While kept quiet, and unexposed to any source of irritation, with attention to the general health, they enjoy a considerable share of rationality and tranquillity; but they are incapable of again mixing with the world, or fulfilling the functions of any office or employment, without risk of complete relapse. These bear a proportion of about one in twenty of the recoveries.

It is evident, from the preceding remarks, that we are more successful *now* in the cure of insanity than formerly. But, from all quarters, we hear of the frequency of *relapses*. Let it be remembered, however, that people who have suffered an attack of any disease—for instance, pneumonia, hepatitis, apoplexy, &c. are more easily thrown into a similar disease, in future, by the same causes, in consequence of certain organs being left in a weakened state. But we do not call these *relapses*: they are *new* attacks, not sequelæ of the former ones. The same observations apply to insanity, and with increased force, since the range of moral and physical causes to which a recovered maniac is exposed, far exceeds that of any other disease, while the patient is generally much less on his guard against the causes which lead to a repetition of the attack.

§ VII. *Pathology.*

" At this word, our readers may fondly entertain the expectation that we can inform them of the long-sought seat of insanity. But, alas! we are far from being able to satisfy them; for dissection has hitherto proved a steril soil, in this respect! The phenomena observed by Willis, Mangetus, Bonetus, Morgagni, Gunz, Meckel, Greding, Vicq. D'Azir, Camper, Chaussier, Gall, &c. have led to nothing but negative and contradictory results. In this investigation, it is of the highest importance to distinguish the *effects* of those diseases which occasioned the *death* of the patient, from those which occasioned the *derangement* of the mind. It is by confounding these together, that there has arisen such contrariety of opinion respecting the pathology of insanity. The details of my researches *post mortem*, would be too long for insertion here; and I shall content myself with presenting the result or conclusions drawn from dissection up to the present day. I do not pretend that these corollaries possess a mathematical accuracy; but I am convinced that they will be found, in general, correct.

" 1. Vices of conformation, in the *skull*, are peculiar to idiotism, fatuity, and cretinism.

" 2. Organic lesions of the *encephalon*, or its envelopes, are only observed in those cases of mental derangement complicated with paralysis, convulsions, epilepsy, or where the patient has sunk under diseases with symptoms analogous to the above.

" 3. Those effusions within the cranium, whether sanguineous or serous, which we sometimes observe, are the *effects* of insanity, or rat' er of those diseases which destroyed the life of the insane.

"4. The changes of structure in the thorax, abdomen, or pelvis, are evidently, in many cases, totally unconnected with the mental derangement. They sometimes indicate the *remote* cause or seat of the malady, but never the *immediate*.

"5. Every kind of organic lesion observed in the bodies of the insane, has been also found in the bodies of those who never evinced a symptom of insanity.

"6. In numerous and accurate dissections of the insane, no alteration whatever from the healthy structure, could be discovered.

"7. Dissection has shown every portion of the encephalon disorganized, suppurated, or otherwise destroyed, without any derangement of the understanding.

"8. From all these premises and facts, we are authorized to infer that, the *brain* being only the *principal* focus of sensibility, there are cases of insanity dependent merely on lesion of its *vital powers:* that there are other cases, where the disease has *not* its seat in the brain, but in various other foci of sensibility, placed in different regions of the body: but, in all cases, that it is a *corporeal* disease, whether the lesion be organic, or only functional."

§ VIII. *Treatment*. There are three general indications to be kept in view, in the treatment of insanity; namely, to remove the physical disorders, check the aberrations of the understanding, and calm the troubled passions.

Among the ancients, helebore was the principal remedy. Accident recommended the shower-bath; and after the circulation of the blood was discovered, venesection was carried to extravagance. In England, the treatment of insanity was long kept a secret; but M. Pinel changed the lot of the insane, broke their chains, and reduced their management to a rational system. Since that period, confidence has been inspired by increased success.

According as the causes of mental derangement are of a general or local, a physical or moral nature, so must the treatment be, general or local; physical or moral. And as there is often a combination of causes, so must there also be a combination of remedial measures. *There is no specific treatment of insanity*. In no two individuals are the functional or organic lesions, the symptoms, and the character of the disease, precisely alike. Hence, each particular case requires a thorough investigation, before the treatment adapted to it, can be fairly laid down. On this account, we must confine ourselves to such general rules of treatment as are more or less adapted to all; leaving the particular application of them to the discretion of the practitioner, after he has studied the individual case.

We have seen that lesion of the sensations, and of the power of association, through defective attention, produced and kept up the hallucination, as well as the perversions of the passions. Every thing, therefore, which can modify the thinking faculty, and control the passions, is an object of the *moral* treatment.

The first question bears on *separation* of the insane from friends and home. On the necessity of this measure, the English, French, and German physicians are unanimously agreed. Willis observed that foreigners were more certainly cured in England than the natives. We find the same thing in France. Strangers, sent to Paris for treatment, are more readily restored to reason, than inhabitants of this city.

"The first effect of separation is to produce a train of new sensations, excited by new objects; and thereby to dissever, more or less, the series of incongruous ideas, from which the patient was previously unable to extricate himself. The consequence is, that he becomes, at this period, more accessible to advice or remonstrance; in short, there is an evident remission of the hallucination, which should be immediately seized on by the physician, for the purpose of gaining the confidence of the patient."

When the insane patient is placed in a proper receptacle, he ought to be under the guidance of one, and *only one* person—and that a physician. Reil, and some others who conceive that there should be a physician, a psycologist, and a moralist, in superintendance on the insane, have no practical knowledge of the subject, and know nothing of the inconvenience, not to say mischief, attending such a triumvirate power. To the medical chief, the domestic attendants must exhibit perfect examples of deference and obedience, for the imitation of the patient; while they themselves wear the appearance of strong delegated power, which will generally render the exercise of it unnecessary, by convincing the insane that all idea of resistance is vain. Interviews with parents and friends must be very cautiously permitted—for the deranged are great children, and children too, whose false ideas have taken a wrong direction.

"Confined thus to a regular life, and an exact discipline, they are naturally led to reflect on this change in their situation; while the necessity of dwelling among, and submitting to the control of strangers, is to them a powerful stimulus to regain their lost freedom and reason."

Some of the insane, on being transferred to a strange place, believe themselves abandoned by their parents and friends. Let the balm of consolation be then poured into their breasts—let them be promised every assistance in once more uniting the bonds of social existence, and they will pass from the gloom of despair to the sunshine of hope. This contrast of sentiment, arising out of the presumed abandonment of friends, and the soothing attention of strangers, produces an internal conflict, from which reason sometimes comes forth victorious. Others imagine that they are conducted to these strange habitations to be delivered up to their enemies, or to be sacrificed. If these fears can be conquered by the kind, affable, and humane conduct of those around them, a recovery may be confidently expected.

"The judicious exercise of the faculties of the alienated, conduces much to recovery. This must be done by directly exciting the *attention* of the insane—by presenting new objects to their contemplation—by giving way, in some measure, to their hallucination. If, by these means, their confidence can be gained, the cure is almost certain."

The *passions* of the insane must be carefully managed. The proud and rebellious passions and emotions must be tamed and kept in subjection, while the timid and melancholy are to be dissipated by encouragement. The art of inspiring *fear* is not of easy management; and ought never to be confided to servants. Many maniacs are incapable of sleeping, while under the depressing influence of fear. This may often be obviated by causing some person to sleep in the room with them, or by leaving a light in their chamber.

It is sometimes proper to substitute a real for an imaginary grievance. Thus a melancholic is devoured with *ennui* in the midst of the pleasures and enjoyments of life. If we withdraw him from his usual habits, and impose on him *real* privations, he will then suffer *real* ennui, which will prove a powerful mean of cure.

"Another believes himself abandoned by his friends, though they are all endeavouring to contribute to his happiness. Deprive him of every testimony of their affection, then he awakes to a sense of his *real* loss, and this is one step towards recovery. Even *physical* sufferings occasionally divert the malady from its mental seat, and relieve the moral evil."

Sometimes the excitive passions, as love, ambition, may be dexterously brought to bear on insanity.

"A melancholic was in a state of great despondency. He was told that he had a lawsuit on hand. The anxiety to defend his suit soon restored his intellectual energy.—A soldier became deranged in mind. He was told that the campaign was about to open. He demanded permission to rejoin his regiment—was forwarded to head quarters, and arrived there in perfect sanity. In short, the examples are innumerable, of the vast influence of our moral over our physical nature. But the character, disposition, and history of each individual, must be carefully studied, in order to manage the moral treatment with success. Music, well managed, acts with considerable power both on the moral and physical frame. There should be but few instruments, the music placed at a distance, and composed of airs with which the patient was familiar in youth; or which he relished before the mental derangement."

Travelling is a useful remedial measure, in many cases. M. Esquirol has always found the complaint mitigated after a long journey, especially if attended by difficulties and privations, and performed through a strange land, where the attention of the insane was strongly excited.

Travelling is also salutary, in a physical point of view. It rouses the torpid functions, particularly those of the abdominal viscera—conduces to sleep—improves the digestion and the secretions. It is, above all, a useful mean of securing convalescence and recovery. Such are the general indications of the moral treatment of the insane. They have for their final object—to force the *maniac* to live *within* himself—the melancholic *without* himself.—" Ils ont pour but d'obliger le maniac à vivre *en lui-mene,* tandis qu'il faut faire vivre le melancholique *hors de lui.*"

*Hygiene and Pharmaceutics.* The insane should have the benefit of free air and ample exercise. Those who are taken ill in a hot country recover best in a cold. Nostalgics, on the other hand, cannot be cured without return to their native soil.

The clothing of the insane, especially of the melancholics, should be warm, so as to encourage and maintain the insensible perspiration. It is an error to imagine that the insane should be deprived of fires, and kept in cold habitations. The administration of food requires much attention. The insane generally devour their victuals with great voracity, and without sufficient mastication. This injures their digestion, and keeps up a source of irritation in the whole line of the alimentary canal. Thirst is also common; therefore, the patient should never be without drink. Wherever there is excitement, the food must be unirritating and small in quantity. Constipation being a very common attendant, the bowels should be sedulously attended to; and all the secretions and excretions promoted by every possible means. For these purposes, exercise in the open air, active or passive, must form a *sine qua non* in the treatment. Every one knows the story of the Scotch farmer, who acquired great celebrity in the cure of the insane. He first took them out to walk with them in his fields, then contrived to quarrel with them, beat them soundly, and afterwards yoked them to the harrow, and made them work like horses!

*Gardening* is an occupation universally applicable to the lower orders of society, and of the greatest use in this complaint. In the upper walks of life, we are obliged to substitute the promenade, music, reading aloud, &c.

To apply *medicines*, with effect, we must first unravel the whole history of the individual and the complaint—ascertain the causes, general and local, which have induced it—the seat, or principal organ affected—whether it is the physical which has first acted on the moral, or the moral on the physical system. What infinite mischief has been done by *Routinists*, in this disease; who, in the sweeping term "insanity," could see but a *single* affection—derangement of the mind!

When we have combated the general dispositions, and the effects of particular morbific causes, and still the mental alienation continues, then we are authorized in attempting more specific means of treatment. Till then we must vary, incessantly vary, our remedial measures, according to the species of insanity with which we are engaged. We shall here confine our remarks to certain medicines which have been deemed heroic in this complaint.

*Baths* have been administered to the insane in every way, and at all temperatures. The warm bath is, upon the whole, the most useful. Patients of a thin, nervous, and irritable constitution, may be kept a considerable time in the water, with great benefit. When there is a strong determination to the brain, the head should be kept encircled with cloths wetted with cold water, while the body is immersed in the warm. Excepting where the patient is young, robust, and complaining of internal heat, the *cold* bath is rarely useful. Where the patient's constitution is debilitated by unmanly excesses, the mere *plonging* in cold salt water is occasionally serviceable. Cold *affusion* from a cock or funnel, is sometimes proper; but requires great caution. It is most adapted to young and unruly patients, with determination to the brain.

At the commencement of insanity, where there is head-ache, redness of eyes and face, throbbing of the carotids, &c. *cold*, locally applied to the head, after leeching or cupping, while irritating pediluvia are also employed, produces salutary effects. The bowels should be kept clear by daily glysters of water alone, or water containing purgative ingredients. This measure is too much neglected in England.

*Emetics* are entitled to considerable rank, especially in melancholia, and where diminished sensibility and torpor prevail. They are dangerous, however, in opposite states of the system, and where there is erythism of any organ or part,

*Purgatives* are exceedingly useful, especially those which act on the biliary secretion, and tend to produce the hæmorrhoidal movement and discharge. Purgatives sometimes produce irritation in the system, and interrupt the free functions of the skin. Whenever this is perceived, they should be alternated with the warm bath. A certain degree of irritation in, and determination to, the abdominal viscera, from purgatives, is, however, very useful; and it is in this way that helebore, gamboge, and other drastic cathartics, have acquired celebrity.

" *Blood-letting* was formerly carried to a most culpable excess. It is indispensible in plethoric subjects, and especially where any long-accustomed hæmorrhage or other evacuation has been suddenly suppressed; but otherwise, *local* blood-letting may almost entirely supersede the lancet in insanity."

Camphor, musk, tonics, antispasmodics, mercury, &c. are all occasionally useful, in individual cases, especially where the *general* indications have been previously attended to; but they are incapable of universal or indiscriminate application.

*Opium.* As maniacs sleep badly, opium and other sedatives have been employed; but they are now proscribed by unanimous consent, as dangerous remedies. Regimen and exercise are the only somniferous measures which can be safely recommended. They are generally successful too.

" Setons, blisters, and counter-irritants, are useful where there has been a metastasis: in monomania, with stupor; in puerperal mania, and sometimes in dementia, or fatuity. It has been recommended to blister, or otherwise excoriate the whole surface of the head. I confess that such measures have not succeeded in my hands. They augment the erythism, torment the patient, increase his irritability, and convince the insane that he is a victim to our cruelty. The swing is occasionally useful in refractory cases."

In respect to the *prevention* of insanity, much might be done by avoiding intermarriages with the insane; by directing the education of youth on stricter principles of religion and morality; by indulging less the caprices and whimsical desires of children, which indulgence unfits them for the rugged journey of life; by not forcing too early the organ of the mind with tasks and exertions beyond its power; by training youth in early regimen and temperance; and, above all, by repressing and controlling each unruly passion as it developes itself from childhood upwards.

In presenting our readers a portrait of this distinguished foreigner's opinions and practices in insanity, we hope and trust that we do a greater service to the cause of medical science, in this country, than by collecting a mass of ephemeral cases, and fleeting speculations, most of which expire in the very cradle of their existence, and few of which have substantial attractions for re-perusal or reference. Under this conviction, we shall make a point of introducing classical and standard articles of Foreign Medical Literature, from time to time, to the english reader, through the medium of this Journal.

---

