# Errata

**Published:** 1820-09-01

**Authors:** 


237, for *materalist,* read *materialist.*

241, line 19 from top, for *irritation,* read *visitation.*

243, line from top 19, dele "*on,*" and read thus:—

"On mankind than all their passions combined."

